# Synthesis of 5-Substituted 3-Amino-1*H*-Pyrazole-4-Carbonitriles as Precursors for Microwave Assisted Regiospecific Syntheses of Pyrazolo[1,5-*a*]Pyrimidines

**DOI:** 10.3390/molecules14010078

**Published:** 2008-12-29

**Authors:** Fawzia Al-Qalaf, Faisal Mandani, Mervat Mohammed Abdelkhalik, Abeer Abdulrahman Bassam

**Affiliations:** 1 Applied Science Department, College of Technological Studies, Public Authority for Applied Education and Training, P. O. Box 42325 Safat, 70654 Kuwait; E-mail: manalabbas@hotmail.com (F. A-O.), sweetheart.2006@hotmail.com (A-A. B.); 2 Chemical Engineering Technology Department, College of Technological Studies, Public Authority for Applied Education and Training, P. O. Box 42325 Safat, 70654 Kuwait

**Keywords:** Oxoalkanonitriles, 2-Aroyl-3-dimethylamino-2-propenenitrile, Pyrazolo[1,5-*a*]pyrimidines, Solvent-free reactions, ^1^H-^15^N HMBC.

## Abstract

A simple route to 3-oxoalkanonitrile **5**, a precursor of the title compounds is described. Reaction of enaminones **2** with hydroxylamine hydrochloride in ethanol yielded aldoximes **3** that were converted readily into **5** in basic medium. This method has been successfully applied with a number of substrates and resulted in excellent yields of the products. Reacting **5** with trichloroacetonitrile afforded 3-amino-2-aroyl-4,4,4-trichloro-2-butenenitriles **6** that condensed with hydrazines to yield 3-amino-1*H*-pyrazole-4-carbonitrile derivatives **8.** Substituted pyrazolo[1,5-*a*]pyridmidines have been prepared with regioselective condensation reactions of **8** with nonsymmetrical dielectrophiles. The structures of compounds obtained were deduced based on ^1^H-NMR, ^1^H-^15^N HMBC- measurements.

## Introduction

**Figure 1:**
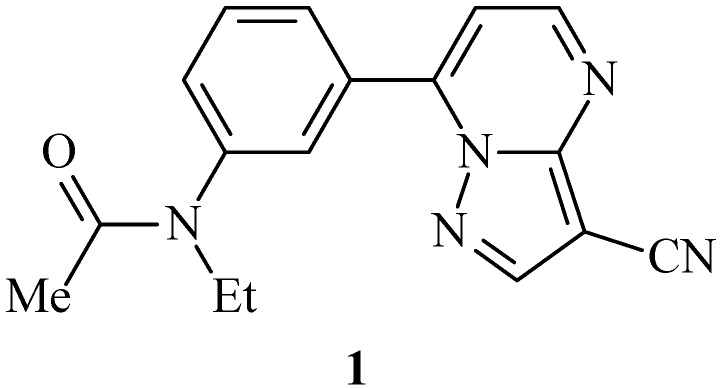
Zaleplon.

Interest in pyrazolo[1,5-*a*]pyrimidine-3-carbonitrile derivatives has been reviewed [1,2,3]. Among them are zaleplon (**1**, Figure 1) analogues, that have been classified as sedative/hypnotic drugs, and as such, are expected to possess considerable biological activity [4,5,6]. With the aim of obtaining compounds possessing the above properties, we examined the condensation of 5-substituted 3-amino-1*H*-4-pyrazolecarbonitriles **8** with bidentate electrophiles and investigated if such compounds would facilitate regioselective syntheses of substituted 7-aryl-pyrazolo[1,5-*a*]pyrimidine-3-carbonitriles that are structurally related to compound **1**. Compound **8a** is readily available from the reaction of 3-oxo-3-phenylpropanenitrile **5a** with trichloroacetonitrile followed by condensation with hydrazine hydrate [7]. This methodology was extended and adapted to the synthesis of several 5-substituted aryl and heteroaryl-pyrazolecarbonitriles using oxoalkanonitriles as precursors. We initially investigated developing a novel route to 3-oxoalkanonitrile derivatives **5**.

## Results and Discussion

**Scheme 1:**
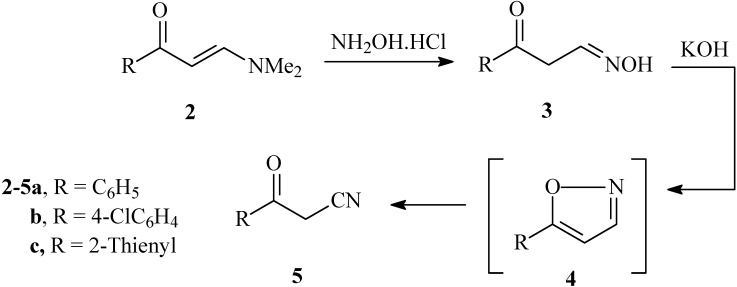
Synthesis of 3-oxo-3-arylpropanenitriles **5a-c** from enaminones **2a-c**.

Recently we reported that the reaction of enaminones **2** with hydroxylamine hydrochloride gave the aldoximes **3** in good yields. These were converted to oxoalkanonitriles **5** *via* treatment with diethyl oxalate in the presence of sodium hydride [8]. We now describe a second more efficient process for the preparation of **5** by addition of a solution of hydroxylamine hydrochloride to enaminones **2a-c** in alcoholic KOH. Such a transformation apparently results *via* initial formation of an isoxazole **4** that then undergoes base catalyzed ring opening furnishing **5a-c** (cf. Scheme 1). 

**Scheme 2:**
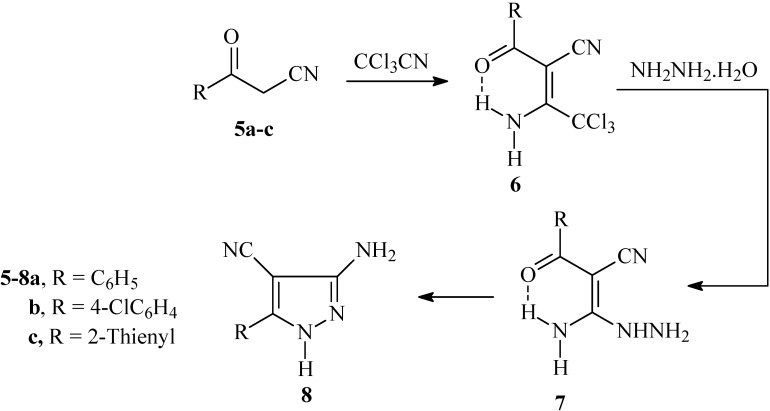
Synthesis of 3-amino-5-aryl-1*H*-4-pyrazolecarbonitrile **8a-c** from 3-oxo-3-arylpropanenitrile **5a-c**.

The oxoalkanonitrile derivatives **5a-c** so obtained were reacted with trichloroacetonitrile to yield adducts **6a-c**. Condensation of these adducts with hydrazine hydrate afforded **7a-c**, that cyclized under reflux in dioxane yielding **8a-c** in good yields (cf. Scheme 2). The doublet splitting of amino group signals observed in the ^1^H-NMR spectra of adducts **6a-c** at ca. δ_H_ = 10 and 12 ppm indicates a non-equivalence of the amino protons, which is probably related to the involvement of one amino proton in an intramolecular H-bond with the carbonyl moiety. Similar features were observed in **7a-c** at ca. δ_H_ = 9 and 10 ppm. There are alternative procedures described in the literature for the preparation of **8a,b** by treating arylmethylenemalononitrile with hydrazine [9,10] or *via* transformation of isothiazoles and isoxazoles into pyrazoles **8a,b** using hydrazines [11]. In the present article, the condensation reaction of 3-amino-1*H*-4-pyrazolecarbonitrile derivatives **8a-c** with enaminone **2c** and enaminonitrile **9,** recently prepared in our laboratory [12], is examined. 

**Scheme 3:**
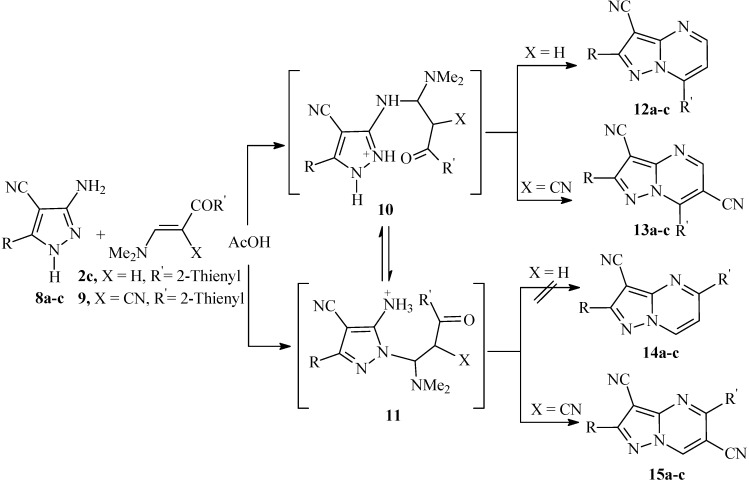
Proposed mechanism for the formation of pyrazolo[1,5-*a*]pyrimidines **12a-c, 13a-c** and **15a-c** in acetic acid.

The site at which nucleophiles attack occurs on 1*H*-3-aminopyrazole derivatives has been a subject of considerable debate in the past [13,14]. Reactions of unsymmetrical 1,3-diketones with 3(5)-aminopyrazoles often lead to the formation of inseparable mixtures of two regioisomeric pyrazolo[1,5-*a*]pyrimidines due to comparable reactivity’s of the two electrophilic centers in the initial diketone. Recently, use of 1,3-dimethyluracil as the electrophile was reported to involve the attack on both endocyclic and exocyclic nitrogen affording either the pyrazolo[1,5-*a*]pyrimidin-5-one or 7-one isomers, depending on the reaction conditions [15], whereas the reaction of 1*H*-3-aminopyrazole with benzylidenemalononitrile [16] and with 1-arylbutane-1,3-diones [17] are established to involve exocyclic amino group. It is thought that there is an equilibrium between possible initial attack at the ring nitrogen and the exocyclic amino group. Structures of the products resulting from reactions of a,b-unsaturated compounds with aminoazoles should be determined in each case, as the outcome of the reactions would be dependent on several factors, including steric consideration, relative basicities and solubility of both isomers in reaction medium. Although it is generally accepted that 3(5)-aminopyrazoles reacts with enaminones to yield the 7-substituted isomers, we noticed that this pattern is not always followed, as the reaction product proved to be dependent on both reaction conditions as well as nature of reagents. In the present article we have found that reaction of **8a-c** with enaminonitrile **2c** in acetic acid at reflux temperature over a long period of time resulted in selective formation of pyrazolo[1,5-*a*]pyrimidines derivatives in good yields (cf. Scheme 3). 

The ^1^H-NMR spectra gave no reliable information on the structure of the products; on the other hand they indicate that only one of the possible isomeric structures, **12** or **14,** are formed, rather than their mixture. The condensation products were assigned structures **12a-c** on the basis of ^1^H-^15^N HMBC measurements. For example compound **12a**, showed chemical shifts for N-7a at δ (^15^N) = 215 ppm, N-4 at δ (^15^N) = 268 ppm. and N-1 at δ (^15^N) = 285 ppm. Cross peak correlations for the coupling of the shielded proton H-6 at δ (^1^H) = 8.05 ppm is observed with N-7a at δ (^15^N) = 215 ppm ^3^*J* (H-6, N-7a), N-4 at δ (^15^N) = 268 ppm ^3^*J* (H-6, N-4) and with N-1 at δ (^15^N) = 285 ppm ^4^*J* (H-6, N-1). Coupling of the deshielded proton at δ (^1^H) = 8.83 ppm with N-7a at δ (^15^N) = 215 ppm ^4^*J* (H-5, N-7a) and with N-4 at δ (^15^N) = 268 ppm ^2^*J* (H-5, N-4) are also observed. Alternative structure **14** would show coupling of the deshielded H-7 proton to be with N-1 in the spectrum at δ (^15^N) = 285 ppm. The correlations in the ^1^H-^15^N HMBC measurements for compounds **12b,c** showed similar coupling correlations as **12a**. Reaction of 3-amino-5-aryl-1*H*-4-pyrazolecarbonitriles **8a-c** with enaminonitrile **9** (X = CN) in acetic acid under the same reaction conditions afforded a mixture of (5)7-substituted pyrazolo[1,5-*a*]pyrimidine derivatives **13a-c** and **15a-c**, which could not be separated by chromatographical means. Both isomers showed the same molecular weights in LC-MS. Moreover, the ^1^H-NMR spectra of the reaction products of **8a-c** with **9** showed two singlets for two deshielded protons at ca. δ_H_ = 9.0 and 9.3 ppm. Based on these data, it is concluded that the compounds obtained are isomeric mixtures of **13** and **15,** whose ratio (approximately 1:3) was estimated by integration of the deshielded protons in the corresponding ^1^H-NMR spectra. Apparently, in AcOH where the aminopyrazoles are most likely protonated, both adducts **10** and **11** are formed, as outlined in Scheme 3. Competing cyclization leading to the 5-isomer would also be possible and mixtures are thus formed.

**Scheme 4:**
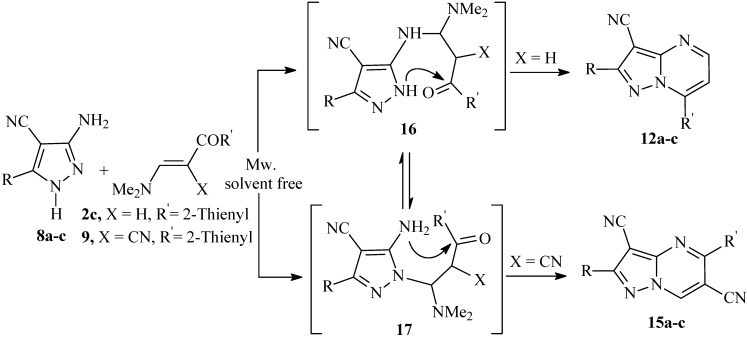
Cyclocondensation reaction of **8a-c** with **2c** and **9** by heating in a direct beam microwave oven and solvent free conditions resulted in selective formation of pyrazolo[1,5-*a*]pyrimidines **12a-c** and **15a-c**.

On the other hand, reaction of 3-amino-5-aryl-1*H*-4-pyrazolecarbonitriles **8a-c** with enaminonitrile **9** (X = CN) by heating in a direct beam microwave oven and under solvent free conditions proceeded regiospecifically to yield the 5-substituted pyrazolo[1,5-*a*]pyrimidine-3-carbonitrile derivatives **15a-c** in good yield. The addition reaction occurs in a manner different to the formation of the 7-substituted pyrazolo[1,5-*a*]pyrimidine derivatives, as outlined in Scheme 4. The structure **15** of the products obtained was assigned on the basis of ^1^H-^15^N HMBC measurements. The major discrepancy between the two isomers **13** and **15** was the cross peak correlation observed between the deshielded proton at δ (^1^H) = 9.06 ppm with N-1 at δ (^15^N) = 283 ppm, ^3^*J* (H-7, N-1). Alternate structure **13** in which the deshielded proton is on C-5, would not show this correlation and thus structure **13** could be ruled out. There is no doubt that adducts at both exocyclic and endocyclic nitrogen atoms occur, but in this case, the latter cyclize more readily into the 5-substituted pyrazolo[1,5-*a*]pyrimidines derivatives thus shifting the equilibrium. It should be noted that the isomers **12a-c** were also obtained as sole products when the reactions of **8a-c** with **2c** was carried out by heating in a direct beam microwave oven and under solvent free conditions. 

The behavior of **8a-c** with **9** under microwave irradiation may be attributed to the formation of hot spots that affect the reaction selectivity due to the increase in heating rate. This may lead to the formation of thermodynamically stable products in preference to the kinetic ones [18,19]. One can thus conclude that this solvent-free reactions proceeds in a regiospecific fashion by the relative reactivity of exocyclic nitrogen and ring nitrogen atoms.

## Experimental

### General

Melting points were determined on a Shimadzu-Gallenkamp apparatus and are uncorrected. Microwave mediated chemistry was conducted in heavy-walled Pyrex tubes fitted with PCS cap. and performed with a single mode cavity Explorer Microwave Synthesizer (CEM Corporation, NC, USA), producing continuous irradiation and equipped with simultaneous external air-cooling system. Elemental analyses were obtained on a LECO CHNS-932 Elemental Analyzer. ^1^H-NMR spectra were obtained in DMSO-*d_6_* on a Bruker DPX 400 MHz superconducting spectrometer in DMSO-d_6_ with TMS as an internal standard. Two-dimensional NMR spectra were determined on Bruker Avance II 600 MHz superconducting spectrometer in DMSO-d_6_ and FT-IR measurements were recorded in KBr disks on a Perkin Elmer 2000 FT-IR system. Mass spectrometric analyses were recorded on a VG-Autospec-Q high performance tri-sector GC/MS/MS.

### General procedure for the preparation of compounds ***5a-c***

A mixture of each enaminone **2a-c** (10 mmol) and NH_2_OH.HCl (0.69 g, 10 mmol) in EtOH (30 mL), was added a solution of KOH (5.60 g, 10 mmol) in H_2_O (8 mL). The reaction mixture was heated under reflux and deemed completed when the yellow color of the solution changed to brown (in 30-60 min.). The reaction mixtures were then poured onto water and neutralized with HCl. The solid products so obtained were collected by filtration and crystallized from benzene.

*3-Oxo-3-phenylpropanenitrile* (**5a**): White crystals, yield (89 %, 1.30 g); mp 79-81 ^o^C (Lit. [12,13] mp. 80-82); IR (cm^-1^): 2255 (CN) and 1689 (CO); MS m/z (M)^+^ = 145; ^1^H-NMR: d = 4.78 (s, 2H, CH_2_), 7.57 (t, 2H, *J* = 6.8 Hz, Ph-H), 7.72 (t, 1H, *J* = 7.4 Hz, Ph-H), 7.94 (d, 2H, *J* = 6.8 Hz, Ph-H); Anal. calcd. for C_9_H_7_NO: (145.16): C, 74.47; H, 4.86; N, 9.65. Found: C, 74.50; H, 4.75; N, 9.57.

*3-(4-Chlorophenyl)-3-oxopropanenitrile* (**5b**): Yellow crystals, yield (86 %, 1.54 g); mp 126-128 ^o^C (Lit. [12,13] mp. 128 ^o^C); IR (cm^-1^): 2255 (CN) and 1680 (CO); MS m/z (M)^+^ = 179; ^1^H-NMR: d = 4.75 (s, 2H, CH_2_), 7.60 (d, 2H, *J* = 8.1 Hz, arom-H), 7.75 (d, 2H, *J* = 8.3 Hz, arom-H); Anal. calcd. for C_9_H_6_Cl NO: (179.01): C, 60.19; H, 3.37; N, 7.80. Found: C, 59.87; H, 3.30; N, 7.67.

*3-Oxo-3-(2-thienyl)propanenitrile* **5c**: Yellow crystals, yield (88 %, 1.32 g); mp 112-114 ^o^C (Lit. [12,13] mp. 110-112 ^o^C); IR (cm^-1^): 3243 (NH_2_), 2255 (CN) and 1666 (CO); MS m/z (M)^+^ = 151; ^1^H-NMR: d = 4.72 (s, 2H, CH_2_), 7.30 (t, 1H, *J* = 4.4 Hz, thienyl H-4), 7.99 (d, 1H, *J* = 4.8 Hz, thienyl H-3), 8.14 (d, 1H, *J* = 4.8 Hz, thienyl H-5); Anal. calcd. for C_7_H_5_NOS: (151.19): C, 55.61; H, 3.33; N, 9.26; S, 21.21. Found: C, 55.72; H, 3.31; N, 9.28; S, 21.00.

### General procedure for the preparation of compounds ***6a-c***

To a stirred mixture of each of oxoalkanonitrile **5a-c** (10 mmol) in EtOH (20 mL) and in the presence of anhydrous NaOAc (1 g), was added 3,3,3-trichloropropanenitrile (10 mmol, 1.57 g). The resulting mixture was stirred for six hrs at r.t., then evaporated under vacuum to half its volume. The reaction mixture was then poured onto water. The solid product obtained was collected by filtration and crystallized from ethanol. 

*(Z)-3-Amino-2-benzoyl-4,4,4-trichloro-2-butenenitrile* (**6a**): Pale yellow crystals, yield (82 %, 2.36 g); mp 182-184 ^o^C; IR (cm^-1^): 3267 and 3250 (NH_2_), 2210 (CN) and 1613 (CO); MS m/z (M^+^-1) = 288; ^1^H-NMR: d = 7.48 (t, 2H, *J* = 7.8 Hz, Ph-H), 7.57 (t, 1H, *J* = 7.9 Hz, Ph-H), 7.68 (d, 2H, *J* = 7.8 Hz, Ph-H), 9.96 (s, 1H, NH_2_), 11.94 (s, 1H, NH_2_); Anal. calcd. for C_11_H_7_Cl_3_N_2_O: (289.55): C, 45.63; H, 2.44; N, 9.67. Found: C, 45.90; H, 2.41; N, 9.56.

*(Z)-3-Amino-4,4,4-trichloro-2-(4-chlorobenzoyl)-2-butenenitrile* (**6b**): Yellow crystals, yield (76 %, 2.46 g); mp 221-222 ^o^C; IR (cm^-1^): 3260 (NH_2_), 2214 (CN) and 1610 (CO); MS m/z (M^+^-1) = 322; ^1^H-NMR: d = 7.58 (d, 2H, *J* = 8.0 Hz, arom-H), 7.71 (d, 2H, *J* = 8.2 Hz, arom-H), 10.05 (s, 1H, NH_2_), 11.87 (s, 1H, NH_2_); Anal. calcd. for C_11_H_6_Cl_4_N_2_O: (323.99): C, 40.78; H, 1.87; N, 8.65. Found: C, 40.90; H, 2.02; N, 8.80.

*(Z)-3-Amino-4,4,4-trichloro-2-(thiophene-2-carbonyl)-2-butenenitrile* (**6c**): Brown crystals, yield (85 %, 2.50 g); mp 168-170 ^o^C; IR (cm^-1^): 3243 (NH_2_), 2216 (CN) and 1624 (CO); MS m/z (M^+^-1) = 294; ^1^H-NMR: d = 7.25 (t, 1H, *J* = 4.0 Hz, thienyl H-4), 8.01 (d, 1H, *J* = 5.2 Hz, thienyl H-3), 8.17 (d, 1H, *J* = 4.0 Hz, thienyl H-5), 9.96 (s, 1H, NH_2_), 12.01 (s, 1H, NH_2_); Anal. calcd. for C_9_H_5_Cl_3_N_2_OS: (295.57): C, 36.57; H, 1.71; N, 9.48; S, 10.85. Found: C, 36.25; H, 1.96; N, 9.25; S, 10.50.

### Reaction of 6a-c with hydrazine hydrate for the preparation of ***7a-c***

To each compound **6a-c** (10 mmol), excess hydrazine hydrate (3 mL) was added and stirred for 3 min. (exothermic reaction). The reaction mixture is then allowed to cool to ambient temperature. During time a precipitate is formed that was filtered off and recrystallized from ethanol. 

*(E)-3-Amino-2-benzoyl-3-hydrazino-2-propenenitrile* (**7a**): This compound was obtained as white crystals, yield (93 %, 1.90 g); mp 143-145 ^o^C; IR (cm^-1^): 3444, 3345 and 3209 (NH and NH_2_), 2182 (CN) and 1656 (CO); MS m/z (M)^+^ = 202; ^1^H-NMR: d = 4.72 (s, 2H, NH_2_), 7.38-7.46 (m, 3H, arom. H), 7.48 (br s, 1H, NH), 7.58 (d, 2H, *J* = 7.8 Hz, arom-H), 8.51 (s, 1H, NH_2_), 9.69 (s, 1H, NH_2_); Anal. calcd. for C_10_H_10_N_4_O: (202.21): C, 59.40; H, 4.98; N, 27.71. Found: C, 59.36; H, 5.2; N, 27.95.

*(E)-3-Amino-2-(4-chlorobenzoyl)- 3-hydrazino-2-propenenitrile* (**7b**): Beige crystals, yield (89 %, 2.10 g); mp 238-240 ^o^C; IR (cm^-1^): 3387, 3350 and 3305 (NH and NH_2_), 2185 (CN) and 1646 (CO); MS m/z (M)^+^ = 236; ^1^H-NMR: d = 4.73 (s, 2H, NH_2_), 7.48 (d, 2H, *J* = 8.0 Hz, arom-H), 7.53 (br s, 1H, NH), 7.59 (d, 2H, *J* = 8.0 Hz, arom-H), 8.54 (s, 1H, NH_2_), 9.97 (s, 1H, NH_2_); Anal. calcd. for C_10_H_9_ClN_4_O: (236.66): C, 50.75; H, 3.83; N, 23.67. Found: C, 50.81; H, 3.92; N, 23.38.

*(Z)-3-Amino-3-hydrazino-2-(2-thienylcarbonyl)-2-propenenitrile* (**7c**): Beige crystals, yield (94 %, 1.95 g); mp 175-177 ^o^C; IR (cm^-1^): 3449, 3331 and 3217 (NH and NH_2_), 2189 (CN) and 1659 (CO); MS m/z (M)^+^ = 208; ^1^H-NMR: d = 4.72 (s, 2H, NH_2_), 7.14 (t, 1H, *J* = 4.0 Hz, thienyl H-4), 7.55 (br s, 1H, NH), 7.76 (d, 1H, *J* = 4.8 Hz, thienyl H-3), 7.90 (d, 1H, *J* = 4.2 Hz, thienyl H-2), 8.54 (s, 1H, NH_2_), 9.98 (s, 1H, NH_2_); Anal. calcd. for C_8_H_8_N_4_OS: (208.24): C, 46.14; H, 3.87; N, 26.90; S, 15.40. Found: C, 45.89; H, 4.09; N, 26.59; S, 15.25.

### General procedure for the preparation of compounds ***8a-c***

Each compound **7a-c** (10 mmol) was refluxed in dioxane (20 mL) for 30 min. then left to cool at r.t. The target compounds separated as crystals that were collected by filtration and crystallized from the appropriate solvent. 

*3-Amino-5-phenyl-1H-4-pyrazolecarbonitrile* (**8a**): Buff crystals from dioxane, yield (93 %, 1.71 g) mp 200-202 ^o^C; IR (cm^-1^): 3348, 3303 (NH_2_), 3193 (NH) and 2230 (CN); MS m/z (M)^+^ = 184; ^1^H- NMR: d = 6.50 (s, 2H, NH_2_), 7.41-7.46 (m, 3H, arom. H), 7.80 (d, 2H, *J* = 7.2 Hz, arom-H), 12.16 (br s, 1H, NH); Anal. calcd. for C_10_H_8_N_4_: (184.20): C, 65.21; H, 4.38; N, 30.42. Found: C, 65.03; H, 4.57; N, 30.12.

*3-Amino-5-(4-chlorophenyl)-1H-4-pyrazolecarbonitrile* (**8b**): Brownish crystals from dioxane, yield (95 %, 2.07 g); mp 218-220 ^o^C; IR (cm^-1^): 3348, 3302 (NH_2_), 3137 (NH) and 2223 (CN); MS m/z (M)^+^ = 218; ^1^H-NMR: d = 6.40 (s, 2H, NH_2_), 7.53 (d, 2H, *J* = 8.4 Hz, arom-H), 7.80 (d, 2H, *J* = 8.4 Hz, arom-H), 12.06 (br s, 1H, NH); Anal. calcd. for C_10_H_7_ClN_4_: (218): C, 54.93; H, 3.23; N, 25.62. Found: C, 55.07; H, 3.46; N, 25.54.

*3-Amino-5-(2-thienyl)-1H-4-pyrazolecarbonitrile* (**8c**): Beige crystals from *n*-propanol, yield (93 %, 1.76 g); mp 238-240 ^o^C**;** IR (cm^-1^): 3325, 3298 (NH_2_), 3177 (NH) and 2228 (CN); MS m/z (M)^+^ = 190;^1^H-NMR: δ = 6.38 (s, 2H, NH_2_), 7.14 (t, 1H, *J* = 4.4 Hz, thienyl H-4), 7.70 (d, 1H, *J* = 4.4 Hz, thienyl H-3), 7.93 (d, 1H, *J* = 4.2 Hz, thienyl H-2), 12.00 (br s, 1H, NH); Anal. calcd. for C_8_H_6_N_4_S: (190.23): C, 50.51; H, 3.18; N, 29.45; S, 16.86. Found: C, 50.63; H, 3.02; N, 29.57; S, 17.02.

### Reaction of ***8a-c*** with enaminone ***2c*** and with enaminonitrile ***9***

*Procedure A*: a mixture of each of compound **8a-c** (10 mmol) and each of **2c** or **9** (10 mmol) was heated under reflux in AcOH (15 mL) for 4h, during which time a precipitate is formed. The reaction mixture was filtered off and recrystallized from acetone. 

*Procedure B*: a mixture of each of compound **8a-c** (10 mmol) and each of **2c** or **9** (10 mmol) was dissolved in ethanol (2 mL) in a small beaker. The reaction mixture was dried in air and the beaker was put in a domestic microwave oven 290 W and irradiate for 15 min. The progress of the reaction was monitored every 3 min. The product was extracted with acetone (2 x 10 mL). The solvent was removed by distillation under reduced pressure to obtain the crude product that was further recrystallized from the appropriate solvent. 

*2-Phenyl-7-(2-thienyl)pyrazolo[1,5-a]pyrimidine-3-carbonitrile* (**12a**): Yellow crystals from acetone, yield (89 %, 2.68 g); mp 237-239 ^o^C; IR (cm^-1^): 2222 (CN); MS m/z (M)^+^ = 302; ^1^H-NMR: d = 7.45 (t, 1H, *J* = 4.2 Hz, thienyl H-4), 7.62-7.70 (m, 3H, arom. H), 8.06 (d, 1H, *J* = 4.8 Hz, H-6), 8.20 (d, 2H, *J* = 8.0 Hz, arom. H), 8.24 (d, 1H, *J* = 4.2 Hz, thienyl H-3), 8.63 (d, 1H, *J* = 4.2 Hz, thienyl H-5), 8.82 (d, 1H, *J* = 4.8 Hz, H-5); Anal. calcd. for C_17_H_10_N_4_S: (302.35): C, 67.53; H, 3.33; N, 18.53; S, 10.61. Found: C, 67.64; H, 3.47; N, 18.47; S, 10.43.

*2-(4-Chlorophenyl)-7-(2-thienyl)pyrazolo[1,5-a]pyrimidine-3-carbonitrile* (**12b**): Brownish red crystals from ethanol, yield (86 %, 2.88 g); mp 252-254 ^o^C; IR (cm^-1^): 2220 (CN); MS m/z (M)^+^ = 336; ^1^H-NMR: d = 7.36 (t, 1H, *J* = 4.2 Hz, thienyl H-4), 7.66 (d, 2H, *J* = 6.6 Hz, arom. H), 7.99 (d, 1H, *J* = 4.8 Hz, H-6), 8.12 (d, 2H, *J* = 6.6 Hz, arom. H), 8.16 (d, 1H, *J* = 4.2 Hz, thienyl H-3), 8.54 (d, 1H, *J* = 4.2 Hz, thienyl H-5), 8.74 (d, 1H, *J* = 4.8 Hz, H-5); Anal. calcd. for C_17_H_9_ClN_4_S: (336.8): C, 60.62; H, 2.69; N, 16.64; S, 9.52. Found: C, 60.32; H, 3.07; N, 16.87; S, 9.64.

*2,7-Di(2-thienyl)pyrazolo[1,5-a]pyrimidine-3-carbonitrile* (**12c**): Beige crystals from acetone, yield (88 %, 2.71 g); mp 251-253 ^o^C; IR (cm^-1^): 2222 (CN); MS m/z (M)^+^ = 308; ^1^H-NMR: d = 7.34 (t, 1H, *J* = 4.8 Hz, thienyl H-4), 7.45 (t, 1H, *J* = 4.8 Hz, thienyl H-4), 7.91 (d, 1H, *J* = 4.8 Hz, thienyl H-3), 7.96 (d, 1H, *J* = 4.8 Hz, thienyl H-3), 8.03 (d, 1H, *J* = 5.2 Hz, H-6), 8.25 (d, 1H, *J* = 4.8 Hz, thienyl H-5), 8.60 (d, 1H, *J* = 4.8 Hz, thienyl H-5), 8.79 (d, 1H, *J* = 5.2 Hz, H-5); Anal. calcd. for C_15_H_8_N_4_S_2_: (308.38): C, 58.42; H, 2.61; N, 18.17; S, 20.80. Found: C, 58.12; H, 2.67; N, 18.09; S, 20.63.

*2-Phenyl-5-(2-thienyl)pyrazolo[1,5-a]pyrimidine-3,6-dicarbonitrile* (**15a**): Grey crystals from acetone, yield (88 %, 2.87 g); mp 291-293 ^o^C; IR (cm^-1^): 2226 (CN); MS m/z (M)^+^ = 327; ^1^H-NMR: d = 7.46 (t, 1H, *J* = 4.2 Hz, thienyl H-4), 7.56-7.60 (m, 3H, arom. H), 8.12 (d, 2H, *J* = 8.2 Hz, arom. H), 8.39 (d, 1H, *J* = 4.2 Hz, thienyl H-3), 8.67 (d, 1H, *J* = 4.2 Hz, thienyl H-5), 9.09 (s, 1H, H-7); Anal. calcd. for C_17_H_10_N_4_S: (327.36): C, 66.04; H, 2.77; N, 21.39; S, 9.79. Found: C, 66.25; H, 2.96; N, 21.17; S, 10.03.

*2-(4-Chlorophenyl)-5-(2-thienyl)pyrazolo[1,5-a]pyrimidine-3,6-dicarbonitrile* (**15b**): Brown crystals from acetone, yield (85 %, 3.06 g); mp 294-295 ^o^C; IR (cm^-1^): 2230 (CN); MS m/z (M)^+^ = 361; ^1^H- NMR: d = 7.46 (t, 1H, *J* = 4.2 Hz, thienyl H-4), 7.67 (d, 2H, *J* = 8.2 Hz, arom. H), 8.08 (d, 2H, *J* = 8.2 Hz, arom. H), 8.39 (d, 1H, *J* = 4.2 Hz, thienyl H-3), 8.65 (d, 1H, *J* = 4.2 Hz, thienyl H-5), 9.08 (s, 1H, H-7); Anal. calcd. for C_17_H_9_ClN_4_S_:_ (361.81): C, 59.75; H, 2.23; N, 19.36; S, 8.86. Found: C, 59.46; H, 2.49; N, 19.19; S, 8.82.

*2,5-Di(2-thienyl)pyrazolo[1,5-a]pyrimidine-3,6-dicarbonitrile* **15c**: Brown crystals from acetone, yield (87 %, 2.89 g); mp 303-304 ^o^C; IR (cm^-1^): 2227 (CN); MS m/z (M)^+^ = 333; ^1^H-NMR: d = 7.27 (t, 1H, *J* = 4.8 Hz, thienyl H-4), 7.45 (t, 1H, *J* = 4.8 Hz, thienyl H-4), 7.87 (d, 1H, *J* = 4.8 Hz, thienyl H-3), 7.92 (d, 1H, *J* = 4.8 Hz, thienyl H-3), 8.40 (d, 1H, *J* = 4.8 Hz, thienyl H-5), 8.63 (d, 1H, *J* = 4.8 Hz, thienyl H-5), 9.06 (s, 1H, H-7); Anal. calcd. for C_15_H_8_N_4_S_2:_ (333.39): C, 57.64; H, 2.12; N, 21.01; S, 19.24. Found: C, 57.42; H, 2.43; N, 20.85; S, 19.57.
